# Prediction of Postoperative Clinical Outcomes in Resected Stage I Non-Small Cell Lung Cancer Focusing on the Preoperative Glasgow Prognostic Score

**DOI:** 10.3390/cancers12010152

**Published:** 2020-01-08

**Authors:** Joerg Lindenmann, Nicole Fink-Neuboeck, Valentin Taucher, Martin Pichler, Florian Posch, Luka Brcic, Elisabeth Smolle, Stephan Koter, Josef Smolle, Freyja Maria Smolle-Juettner

**Affiliations:** 1 Division of Thoracic and Hyperbaric Surgery, Department of Surgery, Medical University of Graz, 8036 Graz, Austria; nicole.neuboeck@medunigraz.at (N.F.-N.); valentin.taucher@medunigraz.at (V.T.); freyja.smolle@medunigraz.at (F.M.S.-J.); 2 Division of Oncology, Department of Internal Medicine, Medical University of Graz, 8036 Graz, Austria; martin.pichler@medunigraz.at (M.P.); florian.posch@medunigraz.at (F.P.); 3 Division of Cancer Medicine, Department of Experimental Therapeutics, The University of Texas MD Anderson Cancer Center, UTHealth, Texas A&M College of Medicine, Houston, TX 77030, USA; 4 Center for Biomarker Research in Medicine (CBmed), Medical University of Graz, 8036 Graz, Austria; 5 Diagnostic and Research Institute of Pathology, Medical University of Graz, 8036 Graz, Austria; luka.brcic@medunigraz.at; 6 Division of Pulmonology, Department of Internal Medicine, Medical University of Graz, 8036 Graz, Austria; elisabeth.smolle@medunigraz.at; 7 Division of Vascular Surgery, Department of Surgery, Medical University of Graz, 8036 Graz, Austria; stephan.koter@medunigraz.at; 8 Institute of Medical Informatics, Statistics and Documentation, Medical University of Graz, 8036 Graz, Austria; josef.smolle@medunigraz.at

**Keywords:** Non-small cell lung cancer, early tumor stage, operation, C-reactive protein, Glasgow Prognostic Score, prognosis

## Abstract

**Background:** The Glasgow Prognostic Score (GPS), which consists of albumin and C-reactive protein (CRP), may predict overall survival (OS) in cancer patients. The aim of this retrospective analysis was to evaluate the clinical impact of the preoperative GPS on patients with resected early stage non-small cell lung cancer (NSCLC). **Methods:** 300 patients with curatively resected stage I NSCLC were followed-up for OS, recurrence-free survival (RFS), cancer-specific survival (CSS), and death from other causes. **Results:** 229 patients (76%) had a preoperative GPS of 0, and 71 (24%) a GPS ≥ 1. The three-year probabilities of RFS, OS, CSS, and death from other causes were 81%, 84%, 88%, and 96% in patients with GPS = 0, and 79%, 74%, 91%, and 82% in patients with a GPS ≥ 1, respectively. GPS ≥ 1 was significantly associated with a higher risk of death from other causes (*p* = 0.022), serving as an independent predictor of death from other causes (*p* = 0.034). Pathologically elevated CRP levels (CRP > 5 mg/L) were found in 91 patients (30%). The mean CRP level was 7.88 ± 15.80 mg/L (0.5–135.6 mg/L). Pre-treatment CRP level was significantly associated with coronary heart disease (*p* < 0.0001), histology (*p* = 0.013), tumor size (*p* = 0.018), tumor stage (*p* = 0.002), and vascular invasion (*p* = 0.017). **Conclusion:** The preoperative GPS predicts adverse survival outcomes in patients with resected stage I NSCLC.

## 1. Introduction

Lung cancer represents one of the most aggressive types of cancer globally. Non-small cell lung cancer (NSCLC) is the most common histologic subtype of primary lung cancer [1]. Despite continuous progress in surgery, chemotherapy, radiation therapy, immune and targeted therapy, the prognosis of patients suffering from lung cancer still remains poor, especially in those with advanced tumor disease. Among patients with operable NSCLC, those with stage I disease have a comparably favorable prognosis with five-year survival rates of about 60–80%, as compared to those patients with stage IIIA disease in which five-year survival rates are only around 30% [2]. Nevertheless, approximately 20–30% of patients with early stage NSCLC will experience tumor recurrence despite adequate stage-based surgical treatment in curative intent [3].

In this context, accurate prognostication of long-term clinical outcome after surgery is of utmost importance for identifying patients at higher risk of recurrence and death, in particular in patients with early stage NSCLC after curative resection in whom further adjuvant treatment is currently not recommend [4]. Currently, the TNM staging system of the International Union Against Cancer (UICC) has been widely used to predict prognosis according to the local, regional, and distant extent of the tumor. However, some authors highlighted that the currently used TNM staging system might insufficiently capture patient prognosis. Importantly, it has been shown that recurrence and survival outcomes even differ within patients of the same tumor stage and after administration of the same treatment [5,6].

In recent years, the impact of chronic inflammatory reaction on the development of cancer has been investigated. In this regard, C-reactive protein (CRP) has shown to serve as a pivotal biological agent. It is a matter of common knowledge that pathologically elevated CRP levels act as risk factors for several occlusive vascular diseases. Moreover, patients with (locally) advanced cancer are mostly associated with considerably increased plasmatic levels of CRP, which may confirm the closed relationship between cancer and chronic inflammation [7,8]. Up to now, several scientific inquiries have extensively documented the intimate relationship between chronic inflammatory response and the development and progression of cancer [9,10,11].

For this purpose, different inflammatory biomarkers have been extensively studied, and inflammation-based scoring indices have been designed in order to facilitate accurate prognostication of risk in cancer patients. The Glasgow Prognostic Score (GPS), which is based on serum albumin and C-reactive protein (CRP) levels, represents one of the most commonly investigated scoring systems usable for several types of cancer [12]. An elevated GPS has shown to be associated with poor survival for both inoperable as well as resectable cancer, including gastro-esophageal cancer [7,8], colo-rectal cancer, pancreatic cancer, liver cancer, breast cancer, renal cancer, and lung cancer, respectively [12].

In the past few years, several studies have reported that an elevated GPS predicts outcome in different stages of NSCLC [5]. The vast majority of these reports investigated the prognostic impact of the GPS in advanced lung cancer [5,6,13,14,15]. However, in the case of early stage NSCLC, only few data are available, in particular for stage I disease [16,17].

Therefore, the aim of this study was to validate the clinical impact of this preoperative score in a large population of patients with curatively resected stage I NSCLC. Particular attention was paid to dissecting the associations of the GPS with specific sub-outcomes, including overall survival (OS), recurrence-free survival (RFS), cancer-specific survival (CSS), and death from other causes.

## 2. Results

### 2.1. Analysis at Baseline

The GPS could be calculated for all 300 included patients. Two hundred twenty-nine patients (76.3%) had GPS 0, 68 (22.7%) had GPS 1, and only 3 (1%) had GPS 2. The mean albumin level for all 300 patients was 4.29 ± 0.49 (range: 2.3–5.4) g/dL, and the mean plasma CRP level was 7.88 ± 15.80 (range: 0.5–135.6) mg/L, respectively (Table 1). The mean GPS was 0.2.

Patients with a GPS > 0 had a significantly higher prevalence of adverse characteristics. Coronary heart disease, peripheral arterial occlusive disease, alcohol abuse, and higher tumor stage as well as vascular invasion were all more prevalent in patients with a GPS > 0 (χ^2^ test: *p* < 0.05). Concerning other laboratory parameters, patients with a GPS > 0 had higher leukocyte numbers, a higher proportion of neutrophils, a lower proportion of lymphocytes, and higher counts of thrombocytes (*t*-Test: *p* < 0.05).

### 2.2. Prospective Analysis—Outcome

Median follow-up time was 3.2 years (range: 6–10.3 years). More than 75% of the patient population was followed at least 1.8 years, and more than 25% of patients were followed for more than 5.4 years. At the end of follow-up, 59 patients (19.7%) had developed tumor recurrence and 73 patients (24.3%) had died. Of these 73 deaths, 38 were attributed to disease progression and 35 to other causes (Table 1). Other causes of death were cardiac decompensation (*n* = 11), respiratory failure (*n* = 6), postoperative pneumonia (*n* = 5), renal failure (*n* = 4), multiple organ failure (*n* = 3), sepsis (*n* = 3), cerebral hemorrhage (*n* = 2), and hepatic failure (*n* = 1), respectively. However, 30 day mortality was only 1%.

For the whole cohort, the 1- and 5-year OS survival estimates were 95% and 73%. The 1- and 5-year RFS probabilities were 93% and 72%. The respective values for CSS were 98% and 84%, and for non-cancer-related survival 97% and 87%.

### 2.3. Predictors of Outcome

In univariate analysis, poor overall survival was associated with male sex, high ASA scores (American Society of Anesthesiologists physical status classification system), peripheral vascular occlusive disease, and advanced tumor grade. Poor CSS was not associated with any of the parameters, while poor non-cancer-related survival was associated with male sex, height, ASA, renal insufficiency, peripheral vascular occlusive disease, and high CRP levels. Again, RFS was not associated with any of the parameters.

### 2.4. Glasgow Prognostic Score (GPS)

Twenty-one of our patients (7%) had hypalbuminemia (<3.5 g/dL), and 53 (18%) had CRP >10 mg/L. Since only three patients had a GPS = 2 (Table 1), only two groups of GPS values were taken into account: GPS = 0 and GPS > 0, respectively. GPS > 0 was statistically associated with larger postoperative tumor size (χ^2^ test: *p* = 0.031), higher tumor stage (*p* = 0.011), coronary heart disease (*p* = 0.004), alcohol consumption (*p* = 0.033), and vascular invasion (*p* = 0.041). A detailed overview is given in Table 2.

Furthermore, GPS > 0 was related to albumin (*p* > 0.0001), CRP (*p* < 0.0001), higher neutrophil counts (*t*-Test: *p* = 0.0019), lower lymphocyte counts (*p* = 0.0001), and higher thrombocyte counts (*p* = 0.0017).

**Figure 1:**
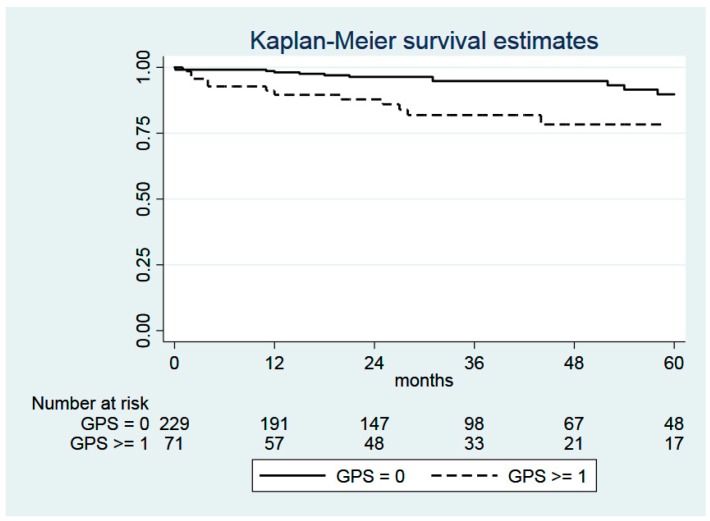
Kaplan–Meier curves comparing the non-cancer-related survival of patients with GPS = 0 (*n* = 229) and with GPS ≥1 (*n* = 71). Log-rank test: χ^2^ = 5.81, *p* = 0.0159. Abbreviation: GPS, Glasgow Prognostic Score.

GPS > 0 was not associated with OS (log-rank test: *p* = 0.1098), RFS (*p* = 0.5133), and CSS (*p* = 0.9446), but it was associated with non-cancer-related survival (*p* = 0.0159; Figure 1).

When Fine and Gray analysis was applied with cancer-related death as a competing risk, non-cancer-related survival was, again, significantly related to GPS > 0 (*p* = 0.022).

In a multivariable, stepwise forward analysis, GPS > 0 (*p* = 0.034) and ASA (*p* = 0.004) were significantly related to non-cancer-related survival, whereas in OS, only ASA turned out to be statistically significant (*p* = 0.002), and in the case of CSS and RFS, respectively, none of these parameters showed statistical significance.

### 2.5. C-Reactive Protein (CRP)

Preoperative plasma levels of CRP were available in all 300 patients. Pathologically elevated concentrations (CRP > 5 mg/L) were found in 91 patients (30.3%), whereas the vast majority (*n* = 209; 69.7%) had normal CRP levels (≤5 mg/L). The mean CRP level was 7.88 ± 15.80 mg/L, ranging from 0.5 to 135.6 mg/L (Table 1). In univariate analysis the CRP level was significantly associated with coronary heart disease (*p* < 0.0001), histology (squamous cell carcinoma; *p* = 0.013), tumor size (T1a; *p* = 0.018 and T2a; *p* = 0.002), tumor stage (*p* = 0.002), and vascular invasion (*p* = 0.017). The detailed clinico-pathological parameters of all 300 patients in relationship to the preoperative plasma CRP levels are summarized in Table 3.

**Figure 2:**
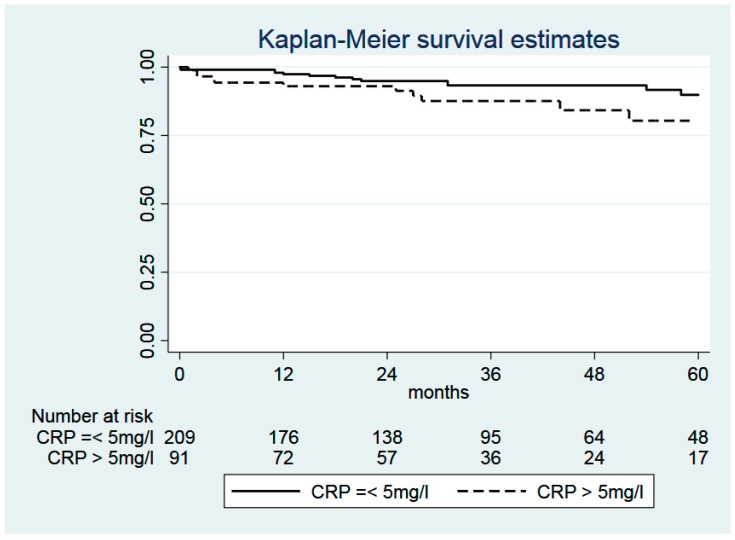
Kaplan–Meier curves comparing the non-cancer-related survival of patients with CRP ≤ 5 mg/L (*n* = 209) and with CRP >5 mg/L (*n* = 91). Log-rank test: χ^2^ = 1.2, *p* = 0.271. Abbreviation: CRP, C-reactive protein.

Enhanced CRP levels (>5 mg/L) were not associated with OS (log-rank test: *p* = 0.148), RFS (*p* = 0.594), CSS (*p* = 0.342), and non-cancer related-survival, respectively (*p* = 0.271, Figure 2).

## 3. Discussion

This study shows that a preoperatively elevated GPS is significantly associated with adverse postoperative long-term outcomes in patients with curatively resected stage I NSCLC. The effect, however, is limited to non-cancer related survival, whereas a relationship with OS, RFS, and CSS could not be detected.

Since the intimate relationship between chronic inflammation and carcinogenesis is documented, several inflammation-based biomarkers and scores have been evaluated for prediction of survival in cancer patients [12]. Over the years, the GPS has evolved as a validated tool that easily captures prognosis in NSCLC based on CRP and albumin levels [7,8]. Primarily this inflammation- and nutrition-based GPS was used to predict survival in patients with advanced lung cancer [5,6,13,14,15,18], whereas a minority of studies investigated the GPS in early-stage disease [16,17]. In contrast to patients with advanced disease representing a heterogenic spectrum, we tried to identify a best possible cohort of stage I patients with homogenic tumor stage without indication for pre- and postoperative therapies.

Basically, the distribution of the GPS differs among different stages of lung cancer. In studies on the GPS focusing on advanced stage III and IV NSCLC patients, the most prevalent score was GPS 1 in 63% of the cases, and a GPS 2 was detected in at least 14% [6,13,15]. Conversely, in studies reporting on stage I and II NSCLC, GPS 0 predominated in 67% and 78% of cases, while GPS 2 was observed in only 7% and 4% of included patients, respectively [16,17]. The findings of the current study confirm these observations.

It has become apparent that any kind of tissue damage induced by infection, trauma, or tumor necrosis will result in a pathological increment of the blood CRP level. The underlying mechanism has been explored as follows. The so-called tumor microenvironment comprises numerous cell types of the inflammatory system that are able to produce specific cytokines (i.e., interleukin-6) in the presence of cancer cells and tumor-related necrosis. By means of the blood flow, interleukin-6 stimulates the hepatocytes to produce acute-phase response proteins, most notably CRP.

After return to the inflammatory tumor microenvironment, CRP is able to dock on the surface of the tumor cells, which are thereby marked for subsequent lysis by the host’s immune system. This induced tumor necrosis results in further production of numerous inflammatory cytokines. As a consequence of those mediators, the cancer cells are stimulated to excrete prostaglandins resulting in further progression of cancer growth. In this context, a vicious circle has been initiated that corroborates the cooperative interaction between the chronic inflammatory reaction and both cancer development and further tumor growth, respectively.

However, there is no consensus regarding the underlying mechanism of CRP increment in patients with (advanced) cancer. It is still a subject of debate whether CRP enhancement is derived from hepatocytes as a consequence of the inflammatory response or directly from malignant tumor cells [7,8,9]. In this regard, increased CRP levels have shown to be associated with tumor growth irrespective of the type of cancer. Patients with advanced stages of cancer exhibit greater tumor load and are accompanied by considerably higher CRP levels than those with early-stage disease [15].

In this context, it has been shown that, among 102 patients with resected NSCLC, the preoperative CRP level was significantly associated with increasing tumor size corresponding to greater tumor load and poorer prognosis [19]. Our data corroborate these findings partially. Increasing CRP and GPS were significantly associated with greater tumor size and tumor stage as demonstrated in literature [20,21,22]. Moreover, a pathologically enhanced CRP level was found to be connected with tumorous vascular invasion corresponding to greater tumor burden and subsequent increased tumor stage (*p* = 0.017).

In the course of meta-analysis, the impact of the CRP value on the prediction of OS among patients with early-stage NSCLC was evaluated [23]. Though it has shown that high pre-treatment CRP levels were associated with poor OS, we were not able to confirm this hypothesis in our present study. In this meta-analysis, ten different articles were investigated showing up with distinctive features. Although the title of this meta-analysis should aim at early-stage NSCLC, 7 out of those 10 studies included patients with different tumor stages ranging from stage I to stage IV. Moreover, the patients underwent different surgical procedures as well as stereotactic body radiation therapy (SBRT), which may also lead to a different outcome regarding OS, RFS, and CSS, respectively.

However, in the course of our literature research we found just one comparable study focusing on the similar endpoint. This study investigated the predictive value of the preoperative GPS in a very small group of 97 elderly patients with resected clinical stage I NSCLC [16]. Even though the number of individuals involved in this study was only one-third of our patient collective, the supposed independent prognostic impact of the preoperative GPS on the postoperative OS could be confirmed.

We could identify several distinctions among a comparable Japanese study, which might explain this different outcome compared to the conclusion of the present study. Although the distribution of stage IA/IB tumors was very similar to our findings (64%/36% vs. 65%/35%), there was a slight difference among the distribution of the preoperative GPS. Miyazaki et al. reported 67% of the evaluated patients had GPS 0, whereas in our study 76% could be detected. Therefore, the corresponding average GPS was 0.2 in our study, compared to 0.4 referred by Miyazaki, which might cause a co-founding influence on the postoperative OS. Concerning the surgical aspects, there were more disparities visible. In our study, complete mediastinal lymph node dissection was done in all patients, whereas only 12.4% had lymph node dissection in the Japanese study. We had 93% with lobectomies compared to 66%, but there were no sublobar resections in our collective, which might influence the postoperative outcome. The median age in our study group was 66 years compared to the elderly collective showing up with 82 years. Thus, the naturally reduced life expectancy might represent a selection bias and could be another explanation of the different outcome of the GPS regarding OS.

However, besides CRP, the plasma albumin level represents the second component of the GPS. In cancer patients, chronic inflammation considerably contributes to both nutritional decline and cachexia resulting in secondary hypalbuminemia [8]. Furthermore, the incidence of hypoalbuminemia is greater among cancer patients with advanced disease compared to those with early-stage disease [7].

In this context, the findings of the current study are favorably in line with these observations. In our population of stage I NSCLC, only 7% of the patients had hypoalbuminemia compared to 32% among those patients with advanced NSCLC [24]. Considering both the low rate of pathologically elevated plasma CRP concentrations and the low incidence of hypoalbuminemia, the distribution of the GPS was thus shifted towards a score of 0 in the vast majority of the cases. Regarding this characteristic distribution, a significant correlation between GPS and postoperative survival could be verified, but only with respect to non-cancer-related survival.

In multivariable modeling, we included several statistically significant predictors of OS from univariable analysis, such as male sex and the preoperative ASA score reflecting patient individual comorbidity. In this analysis, the GPS has shown to be significantly associated with death from other causes after multivariable adjustment, but not RFS, OS, or CSS. This shows that the GPS in stage I NSCLC serves as an independent predictor of non-cancer-related postoperative outcomes.

To the best of our knowledge, this study is the first to additionally investigate the association of the preoperative GPS with specific postoperative sub-outcomes including risk of recurrence, death from cancer, and death from other causes in a cohort limited to stage I NSCLC. Competing risk analysis was used as a statistical method to determine these very endpoints, which clearly showed the different impact of the GPS on death from other causes as well as on death from cancer mentioned above. Importantly, our results show for the first time a strong prognostic relationship between an elevated GPS and an increased risk for death from other causes, whereas no association was found between the GPS and cancer recurrence or death from cancer. The explanation for this finding may be that an elevated GPS in stage I NSCLC is likely more reflective of comorbidity, setting patients at risk of non-cancer-related death, rather than tumor biology and burden. In this respect, we could verify a statistically significant association of an elevated GPS with coronary heart disease (*p* = 0.004) and alcohol (*p* = 0.033). Moreover, we could demonstrate a statistically significant correlation between pathologically elevated CRP levels and coronary heart disease (*p* < 0.0001). These findings have important implications because they suggest that, for the group of patients with stage I NSCLC, an elevated GPS would be best suited as a tool to identify patients that require more intensive management of comorbidity, rather than patients who have a higher risk of recurrence. In this context, the treatment of co-morbidity has to be tailored to the individual needs of each patient and should be performed in addition to the conventional postoperative oncological follow-up in order to decrease lifestyle-related mortality. Considering the intensified statistical evaluation of these special sub-outcomes, the obtained findings are novel within this special field of interest.

Finally, there are some limitations in this study that have to be mentioned. The study was retrospective, observational, and conducted at a single institution. Moreover, we cannot rule out the presence of some residual confounding by factors that were not included in the analysis because they were not collected during data ascertainment.

## 4. Materials and Methods

A total of 334 consecutive patients with pathologic stage I NSCLC who had undergone surgical resection between 2003 and 2015 at a single institution in Central Europe, the Medical University of Graz, Austria, were evaluated retrospectively. Three hundred (90%) of these 334 patients had complete medical data and met the inclusion criteria and were therefore included in this analysis. The inclusion criteria were male and female patients with pathologically confirmed stage I NSCLC after curative resection. Exclusion criteria were, amongst others, tumor stage greater than UICC stage IB and every type of sub-lobar resection (wedge resection, anatomical segmentectomy). In order to avoid statistical bias regarding the correct calculation of the OS, the CSS, and the RFS, patients with documented incidence of a second cancer before or after surgery were excluded too. Patients with ongoing infection or inflammatory reaction usually present with leukocytosis and/or elevated CRP levels, which automatically precludes calculation of the GPS. Therefore, this very collective of patients was excluded too.

At the time of surgery, none of those included patients showed clinical evidence of any inflammatory condition or bacterial infection. Appropriate clinical investigation, laboratory tests, and imaging methods were done to rule out any inflammation, as previously described [7,8].

The patient-specific data were collected prospectively in the database of our hospital and retrospectively extracted for statistical evaluation, as previously described [7,8]. Cancer recurrence and survival outcome data were ascertained from follow-up investigations documented in the center’s electronic health records, by telephone interview with the patients, from their family doctor, or by contacting the local population registry office. This retrospective data analysis was approved by the Local Ethics Committee of the Medical University of Graz, Austria (Nr. 28-316 ex 15/16). Written informed consent from the patients was not required because of the retrospective character of the present study.

Pre-treatment blood samples including CRP and albumin were collected at patient admission to our department before any diagnostic, interventional, or surgical procedure was started. The samples were immediately processed as previously reported [7,8]. Because of the current standards defined by the university clinical institute of medical and chemical laboratory diagnostics, a plasma CRP concentration of more than 5 mg/L was considered pathological. Normal values for albumin were determined from 3.5 to 5.3 g/dL.

The preoperative GPS was constructed as shown in Table 4. Patients with CRP >10 mg/L and albumin <3.5 g/dL were allocated a score of 2. Patients were allocated to a GPS 1 only if CRP was increased or albumin decreased. Patients in whom neither of these abnormalities were present were allocated a score of 0 [7,12,14,16].

Moreover, each patient´s individual performance status and the preoperative ASA surgical risk classification were assessed before surgery (ASA physical status; American Society of Anesthesiologists). All of the 300 included patients underwent pulmonary resection with curative intent and with complete mediastinal lymph node dissection according to the recent oncological guidelines in thoracic surgery [25]. After operation, every patient was routinely referred to our interdisciplinary tumor board. The postoperative tumor staging was determined according to the 7th edition of the TNM staging system of the UICC [26]. Based on the staging results corresponding to UICC stage I, an adjuvant chemotherapy or radiation therapy was not recommended in any of those cases [4]. In every patient, the further postoperative follow-up was done by CT scan of the thorax, the mediastinum, and the abdomen every six months for the first postoperative two years, then annually for the following three years [27].

OS was defined as the time from the date of surgery to the date of death from any cause. RFS was calculated from the date of surgery to the date of diagnosis of tumor recurrence or death from any cause, whichever came first. Risks of cancer recurrence and CSS were determined from the date of surgery to the date of tumor recurrence, or the date of death after tumor recurrence, respectively.

Death from other causes was similarly computed from the date of surgery to the date of death from causes other than tumor recurrence.

### Statistical Analysis

All statistical analyses were performed using Stata (Windows version 15, Stata Corp., Houston, TX, USA). Sample size calculation was done for these 300 cases, with an alpha value of 0.05, and a hazard ratio of a binary covariate of 2.0, considering an event probability of 36%, yielding a power of 0.95 for a Cox analysis. Power analysis showed that more subtle changes may well be overlooked. Continuous variables were summarized as mean and standard deviation, and categorical variables as absolute and relative counts. The association between two categorical variables was assessed with Pearson’s χ^2^ test. Relationships between binary variables and continuous variables were evaluated by Student’s *t*-Test. Time to death from any cause was investigated with Kaplan–Meier estimators, log-rank tests, and Cox proportional hazards models. Time to death from other causes was assessed with death from cancer as competing risk according to Fine and Gray proportional subdistribution hazards models. A *p*-value < 0.05 was considered to indicate statistical significance.

## 5. Conclusions

In conclusion, the preoperative GPS represents an appropriate score for the prediction of non-tumor-related survival after lung cancer surgery in patients with stage I NSCLC.

## Figures and Tables

**Table 1:** Compact summary of the oncological aspects of 300 patients undergoing curative surgery for stage I non-small cell lung cancer (NSCLC). Abbreviations: BMI, body mass index; CRP, C-reactive protein; GPS, Glasgow Prognostic Score; ASA, American Society of Anesthesiologists physical status classification system.

Criterion	Value
Number of patients	300
Age	65.4 ± 10.0 (20–87)
Male/Female	187 (62.3%)/113 (37.7%)
BMI	26.3 ± 4.3 (15.7–9.5)
Death	73 (24.3%)
Death due to tumor	38 (12.7%)
Death not tumor related	35 (11.7%)
Tumor recurrence	59 (19.7%)
Postoperative tumor size	
T1a	116 (38.7%)
T1b	78 (26.0%)
T2	106 (35.3%)
Albumin (g/dL)	4.29 ± 0.49 (2.3–5.4)
CRP (mg/L)	7.88 ± 15.80 (0.5–135.6)
GPS	
0	229 (76.3%)
1	68 (22.7%)
2	3 (1.0%)
ASA	
1	7 (2.3%)
2	101 (33.7%)
3	173 (57.7%)
4	19 (6.3%)
Follow-up time (months)	38.1 ± 28.3 (0–123)
Overall survival	
1 year	94.2% ± 1.4%
3 year	80.8% ± 2.7%
5 year	72.5% ± 3.5%
10 year	29.0% ± 9.2%

**Table 2:** Clinico-pathological parameters of 300 patients with stage I NSCLC after curative resection in regard to the calculated preoperative GPS. Abbreviations: GPS, Glasgow Prognostic Score; BMI, body mass index; COPD, chronic obstructive pulmonary disease; PAD, peripheral arterial disease; CKD, chronic kidney disease; CHD, coronary heart disease; CRP, C-reactive protein.

Criterion	All Cases	GPS 0	GPS ≥ 1	*p* Value
	*n* = 300 (100%)	*n* = 229 (76%)	*n* = 71 (24%)	
Age (years; mean)	66.0 (60.1–72.2)	65.3	68.3	0.243
Weight (kg; mean)	75 (65–85)	75	75	0.995
BMI (kg/m^2^; mean)	26.2 (23.5–28.6)	26.3	26.1	0.865
**Gender**				
Male	187 (62%)	139 (61%)	48 (68%)	0.294
Female	113 (38%)	90 (39%)	23 (32%)	
**Smoking Status**				
Yes	199 (66%)	149 (65%)	50 (70%)	0.404
No	101 (34%)	80 (35%)	21 (30%)	
**COPD**				
Yes	126 (42%)	95 (41%)	31 (44%)	0.745
No	174 (58%)	134 (59%)	40 (56%)	
**PAD**				
Yes	25 (8%)	19 (8%)	6 (8%)	0.967
No	275 (92%)	210 (92%)	65 (92%)	
**CKD**				
Yes	17 (6%)	12 (5%)	5 (7%)	0.566
No	283 (94%)	217 (95%)	66 (93%)	
**CHD**				
Yes	41 (14%)	24 (10%)	17 (24%)	**0.004**
No	259 (86%)	205 (90%)	54 (76%)	
**Alcohol abuse**				
Yes	136 (45%)	96 (42%)	40 (56%)	**0.033**
No	164 (55%)	133 (58%)	31 (44%)	
**Albumin (g/dL)**				**<0.0001**
Albumin < 3.5 g/dL	21 (7%)	0 (0%)	21 (30%)	
Albumin ≥ 3.5 g/dL	279 (93%)	229 (100%)	50 (70%)	
**CRP (mg/L)**				**<0.0001**
CRP ≤ 10 mg/L	247 (82%)	229 (100%)	18 (25%)	
CRP > 10 mg/L	53 (18%)	0 (0%)	53 (75%)	
**Histology**				0.101
Adenocarcinoma	191 (64%)	154 (67%)	37 (52%)	
Squamous cell	95 (32%)	64 (28%)	31 (44%)	
Adenosquamous	2 (1%)	2 (1%)	0 (0%)	
Large cell	2 (1%)	2 (1%)	0 (0%)	
Other	10 (3%)	7 (3%)	3 (4%)	
**Grading**				
G1	55 (18%)	45 (19%)	10 (14%)	0.507
G2	131 (44%)	100 (44%)	31 (44%)	
G3	114 (38%)	84 (37%)	30 (42%)	
**Tumor size**				
T1a	116 (39%)	96 (42%)	20 (28%)	**0.024**
T1b	78 (26%)	61 (27%)	17 (24%)	0.367
T2a	106 (35%)	72 (31%)	34 (48%)	**0.002**
**Tumor stage**				**0.011**
IA	194 (65%)	157 (69%)	37 (52%)	
IB	106 (35%)	72 (31%)	34 (48%)	
**Lymphatic invasion**				
Yes	80 (27%)	58 (25%)	22 (31%)	0.346
No	220 (73%)	171 (75%)	49 (69%)	
**Vascular invasion**				
Yes	28 (9%)	17 (7%)	11 (15%)	**0.041**
No	272 (91%)	212 (93%)	60 (85%)	
**Surgical procedure**				0.721
Lobectomy	279 (93%)	214 (93%)	65 (91%)	
Bilobectomy	8 (3%)	6 (3%)	2 (3%)	
Sleeve Lobectomy	11 (4%)	7 (3%)	4 (6%)	
Pneumonectomy	2 (1%)	2 (1%)	0 (0%)	

**Table 3:** Clinico-pathological parameters of 300 patients with stage I NSCLC after curative resection in regard to the preoperative CRP level. Abbreviations: BMI, body mass index; COPD, chronic obstructive pulmonary disease; PAD, peripheral arterial disease; CKD, chronic kidney disease; CHD, coronary heart disease; CRP, C-reactive protein.

Criterion	All Cases	CRP ≤ 5 mg/L	CRP > 5 mg/L	*p* Value
	*n* = 300 (100%)	*n* = 209 (70%)	*n* = 91 (30%)	
Age (years; mean)	66.0 (60.1–72.2)	64.9	66.3	0.870
Weight (kg; mean)	75 (65–85)	74.8	78.3	0.976
BMI (kg/m^2^; mean)	26.2 (23.5–28.6)	26.0	27.1	0.979
**Gender**				
Male	187 (62%)	124 (59%)	63 (69%)	0.104
Female	113 (38%)	85 (41%)	28 (31%)	
**Smoking Status**				
Yes	199 (66%)	137 (66%)	62 (68%)	0.664
No	101 (34%)	72 (34%)	29 (32%)	
**COPD**				
Yes	126 (42%)	84 (40%)	42 (46%)	0.336
No	174 (58%)	125 (60%)	49 (54%)	
**PAD**				
Yes	25 (8%)	18 (9%)	7 (8%)	0.791
No	275 (92%)	191 (91%)	84 (92%)	
**CKD**				
Yes	17 (6%)	11 (5%)	6 (7%)	0.647
No	283 (94%)	198 (95%)	85 (93%)	
**CHD**				
Yes	41 (14%)	18 (9%)	23 (25%)	**<0.0001**
No	259 (86%)	191 (91%)	68 (75%)	
**Alcohol abuse**				
Yes	136 (45%)	91 (44%)	45 (49%)	0.345
No	164 (55%)	118 (56%)	46 (51%)	
**Albumin (g/dL)**				0.422
Albumin < 3.5 g/dL	21 (7%)	13 (6%)	8 (9%)	
Albumin ≥ 3.5 g/dL	279 (93%)	196 (94%)	83 (91%)	
**Histology**				
Adenocarcinoma	191 (64%)	140 (67%)	51 (56%)	
Squamous cell	95 (32%)	57 (27%)	38 (42%)	**0.013**
Adenosquamous	2 (1%)	1 (1%)	1 (1%)	
Large cell	2 (1%)	2 (1%)	0 (0%)	
Other	10 (3%)	9 (4%)	1 (1%)	
**Grading**				
G1	55 (18%)	44 (21%)	11 (12%)	0.172
G2	131 (44%)	87 (42%)	44 (48%)	
G3	114 (38%)	78 (37%)	36 (40%)	
**Tumor size**				
T1a	116 (39%)	90 (43%)	26 (29%)	**0.018**
T1b	78 (26%)	57 (27%)	21 (23%)	0.446
T2a	106 (35%)	62 (30%)	44 (48%)	**0.002**
**Tumor stage**				
IA	194 (65%)	147 (70%)	47 (52%)	**0.002**
IB	106 (35%)	62 (30%)	44 (48%)	
**Lymphatic invasion**				
Yes	80 (27%)	52 (25%)	28 (31%)	0.289
No	220 (73%)	157 (75%)	63 (69%)	
**Vascular invasion**				
Yes	28 (9%)	14 (7%)	14 (15%)	**0.017**
No	272 (91%)	195 (93%)	77 (85%)	
**Surgical procedure**				
Lobectomy	279 (93%)	195 (93%)	84 (92%)	0.756
Bilobectomy	8 (3%)	4 (2%)	4 (5%)	0.220
Sleeve Lobectomy	11 (4%)	8 (4%)	3 (3%)	0.822
Pneumonectomy	2 (1%)	2 (1%)	0 (0%)	0.349

**Table 4:** Description of the preoperative GPS. Abbreviations: GPS, Glasgow Prognostic Score; CRP, C-reactive protein.

Description	GPS
CRP ≤ 10 mg/L and albumin ≥ 3.5 g/dL	0
CRP ≤ 10 mg/L and albumin < 3.5 g/dL	1
CRP > 10 mg/L and albumin ≥ 3.5 g/dL	1
CRP > 10 mg/L and albumin < 3.5 g/dL	2
